# Anthropocene Imperilment of Ancient Diversity and Evolutionary Potential in Terrestrial Vertebrates

**DOI:** 10.21203/rs.3.rs-7556378/v1

**Published:** 2025-09-25

**Authors:** R. Alexander Pyron, Mario R. Moura, Rauri C. K. Bowie, Selma, F. Brito, Karoline Ceron, Timothy J. Colston, Jacob A. Esselstyn, Jhonny J. M. Guedes, Robert P. Guralnick, Hidalgo V. G. Lima, Arne Ø. Mooers, Matheus T. Moroti, Maria F. Paiva, Matt Pennell, Renata M. Pirani, José Alan S. Souza, João F. R. Tonini, Nathan S. Upham, João Xavier, Walter Jetz

**Affiliations:** The George Washington University; Universidade Federal da Paraiba; University of California, Berkeley; Universidade Federal do Ceará; Universidade Federal do Ceará; George Washington Univeristy; Louisiana State University; Universidade Federal de Goiás; University of Florida; Universidade Federal da Paraíba; Simon Fraser University; Universidade Estadual de Campinas; Universidade Federal do Ceará; Cornell University; University of California, Los Angeles; Universidade Federal da Paraíba; University of Richmond; Arizona State University; Universidade Federal de Santa Catarina; Yale University

## Abstract

The ecological and evolutionary consequences of ongoing extinction episodes remain poorly understood^1^, despite mounting evidence of global biodiversity loss. To assess how human activities are reshaping tetrapod evolution, we estimate species-level extinction probabilities (‘pEX’) over the next ~50–500 years^2,3^ using time-calibrated phylogenies^4–8^, 35 ecological and environmental attributes^9^, and current-day, expert-assessed threats for 33,281 terrestrial vertebrates^2^. We find a critical, divergent association between extinction risk and macroevolutionary patterns^10^: in birds, lizards, and snakes, both evolutionary distinct and rapidly diversifying lineages are most imperiled; while in amphibians and mammals, threat is concentrated in less distinct and slowly radiating groups. Overall, species with high fecundity, intermediate body sizes, and broad geographic ranges are more likely to persist through the ongoing extinction crisis^11–14^. Without intervention, current-day threats alone suggest a 15% decline (~5,000 extinctions) in species richness and a 16% loss in median speciation rate from 0.11 to 0.094 lineages per million years within the next 500 years. Notably, mammals are projected to experience the largest declines in future speciation potential, despite lower overall imperilment than turtles, crocodilians, or amphibians. Across tetrapods, projected evolutionary distinct extinctions are concentrated in tropical regions, whereas faster-radiating lineages face widespread risk across deserts, tropical islands, and temperate zones. These results uncover ecological and evolutionary drivers of the ongoing reorganization of Earth’s biodiversity, underscoring the urgent need for coordinated conservation efforts to preserve both deep evolutionary history and future potential for evolutionary responses.

The long-term ecological and evolutionary impacts of human-driven global change are poorly known^1,15^, despite substantial research on their short-term functional^16^, phylogenetic^17^, and taxonomic^18^ dimensions. Specifically, whether modern extinctions threaten ancient diversity or evolutionary novelty more severely or shift the geography and ecology of speciation and extinction – thus reshaping compound traits and functions – remains largely unknown^10,11,16,19–21^. An initial hurdle is quantifying evolutionary success, typically reflected in either long-term persistence (yielding ancient, distinctive lineages, often with fewer species) or rapid diversification (producing many, often short-lived species)^2,4^. Whether the current biodiversity crisis disproportionately threatens either mode remains unclear^10,17^. Identifying attributes less affected by extinction is equally vital for predicting and mitigating contemporary biodiversity loss^16^, as past selective extinction often eliminated species traits (e.g., large body sizes, specialized niches) that were advantageous in stable periods but detrimental during disturbances, drastically altering post-extinction recovery^22^. Finally, we still do not know which lineages might suffer the greatest losses of ancient phylogenetic diversity and how future evolutionary potential is shifting across clades and geographies due to changes in net diversification rates^1^.

Understanding the ecological and evolutionary consequences of global change has been limited by the lack of species-level data on phylogenetic relationships with branch lengths estimated under a consistent set of prior distributions^23^, comprehensive data for traits and threats^9^, and a robust species-level metric of extinction risk^24^. Building on our earlier work, we present time-calibrated phylogenies for 33,281 tetrapod species spanning amphibians, birds, mammals, and reptiles^4–8^. We pair this with an expanded database^9^ of 35 attributes linked to imperilment^12–14^, including biogeography, climate, population trends, anthropogenic threats, and life- and natural-history traits like diet, reproductive mode, litter size, and longevity (Supplementary Table 1). Combined with a selection of phylogenetic and spatial filters, this represents 188 variables per species, including data from recent extinctions (Late Pleistocene–Holocene) when available.

Using machine learning^25^, we model the complex link^26^ between species’ attributes and their expert-assessed threat status^2,27,28^ (Extended Data Fig. 1–3). Extending previous approaches working with discrete threat categories^2,3^, we uncover a latent multivariate relationship that connects species’ characteristics to a continuous liability of short-term extinction^29,30^. Using the trained models, we developed a measure of extinction probability (pEX; Fig. 1; Methods) projecting the current-day risk over the next ~50–500 years^2,3^. The strongest predictor is range size, while population trends, body size, the number and type of threats and habitats, proximity to urbanization, longevity, and elevation also show strong impacts. The pEX metric captures how extinction risk varies continuously across species, based on their unique combinations of history, geography, and traits (Supplementary Table 2–4) and is validated by observed recent extinction timelines in birds^31^ (Extended Data Fig. 4; Supplementary Table 5).

## Ancient Diversity and Evolutionary Novelty

Both evolutionarily ancient and recently diversifying species face elevated risk across tetrapods, though this relationship varies substantially between clades (Fig. 2, 3; Extended Data Fig. 5, 6). While some studies have suggested a relationship between extinction risk, ancient diversity, and evolutionary novelty in specific groups^5,10,32,33^, our pEX metric provides the first comprehensive evidence across tetrapods. We modeled these relationships using beta regression to link the mean and variance of pEX to Clade^10^, Evolutionary Distinctness^34^ (ED), and Diversification Rate^4^ (DR) as predictors. Notably, pEX exhibits a significant decrease with both ED and DR in amphibians and mammals but increases in birds and lepidosaurs (Extended Data Fig. 7). Consequently, both evolutionary distinct and faster-diversifying birds, lizards, and snakes face higher extinction risk, whereas both less-distinct and slower-diversifying amphibians and mammals tend to be more imperiled. Most species of crocodilians and turtles face high extinction risk regardless of their ED, DR, or other attributes.

The ED and DR metrics show opposing latitudinal gradients globally (Extended Data Fig. 8). While ED decreases with latitude, DR shows an inverse gradient, mirroring recent results^35,36^ for amphibians, birds, and mammals. Similarly, pEX increases with latitude, while also peaking at higher elevations and latitudes, and arid equatorial regions. Tropical islands (e.g., Madagascar, the Caribbean) and the Indo-Malayan realm show high extinction risk and total range rarity, a measure of endemism (Fig. 4a). Correcting for species richness, ED is underrepresented at higher latitudes and arid regions and overrepresented in the tropics, whereas the reverse is true for DR and pEX, and rarity peaks in tropical mountains and major island systems. These patterns mirror hotspots of richness, threat, and phylogenetic diversity^19,27,28^, and support the long-standing hypothesis that lower tropical extinction drive species accumulation^23^, particularly of range-restricted taxa.

Applied to all species, the model projects up to 40% declines in both ED and DR for high latitudes, most island systems (except New Guinea), and the western Palearctic and Indo-Malayan realms (Fig. 4). Disproportionate losses of ED are expected in South American Atlantic forests, arid and dry tropical regions of Africa and the Middle East, and across the central and eastern Palearctic and central Australia. The largest relative decreases in DR are projected for the Nearctic, southern Andes, western Africa and Ethiopia, eastern Australia, and the northeastern Palearctic. A key longitudinal divide emerges in ED and DR imperilment (Fig. 2), where impacts on both metrics are concentrated in the Old-World tropics. In contrast, New World assemblages primarily show impacts on DR in both temperate and tropical regions, apart from polar latitudes and the Atlantic Forest region of Brazil where ED is more imperiled and the Caribbean where both are threatened. Notably, lowland tropical forests in the Amazon, Congo basins, and New Guinea are less affected than most other temperate and tropical regions on both islands and continents.

## Global Geography of At-Risk Traits

**Fig. 1 |:**
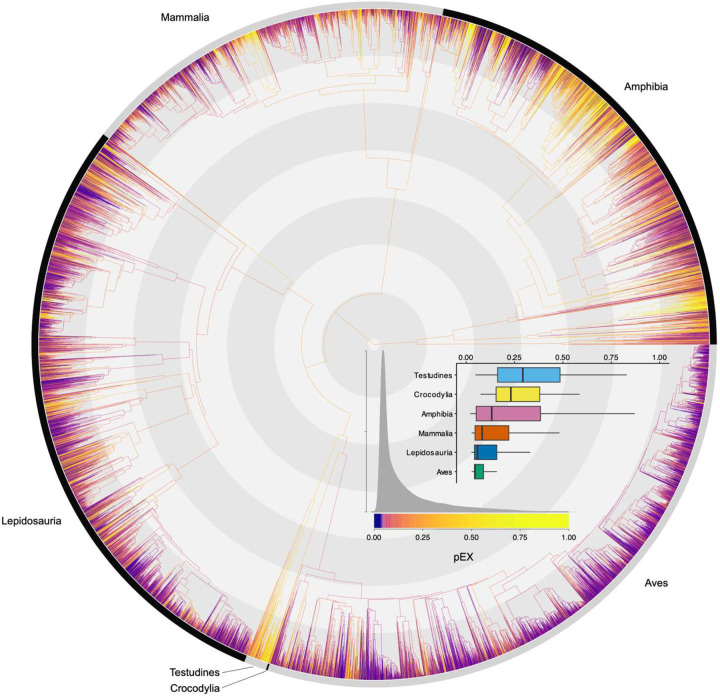
Species-level present-day extinction probabilities across the Tetrapod Tree of Life. Median species-specific pEX values plotted across the tree of 33,281 recent tetrapods. Inset plot shows the median, interquartile range, and 1.5 * IQR for the six major clades. This metric reflects the underlying probability of extinction per species over ~50–500 years derived from the relationship between expert-assessed threat status and 188 variables (Extended Data Fig. 1).

**Fig. 2 |:**
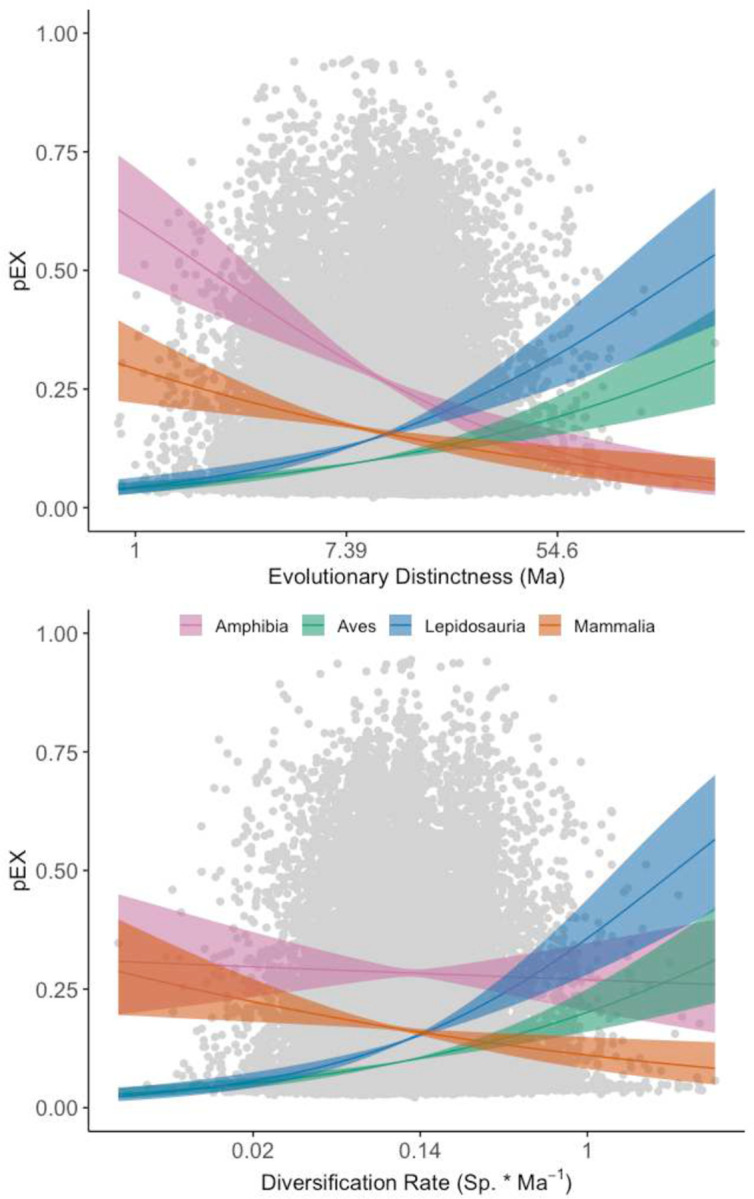
Evolutionary correlates of Anthropocene imperilment. **a, b** Relationship between pEX and Evolutionary Distinctness (ED: Ma) showing positive associations in lepidosaurs and birds and negative associations in amphibians and mammals, and Diversification Rate (DR: Sp. * Ma^−1^) showing positive associations in lepidosaurs and birds and negative associations in amphibians and mammals (Extended Data Fig. 7). We tested the full range of 196 possible models including all interaction terms, with the strongest support (ΔAIC = 102) for the full 48-parameter non-phylogenetic model (see Methods for details; Supplementary Table 6). Model parameters within crocodilians and turtles were not significant and are therefore not shown.

Global change disrupts the fitness landscape for formerly adaptive traits^37^ by shrinking the ecological space viable for both persistence and diversification. The highest pEX values (Fig. 1, Extended Data Fig. 7) are estimated for taxa with ranges < ~22,000 km^2^ (the approximate area of Belize) and body masses >150g (comparable to chipmunks). Binary attributes conferring >5% increases in median pEX are occurrence in tropical and subtropical coniferous or dry broadleaf forests; Oceania; and marine, island, or aquatic environments.

**Fig. 3 |:**
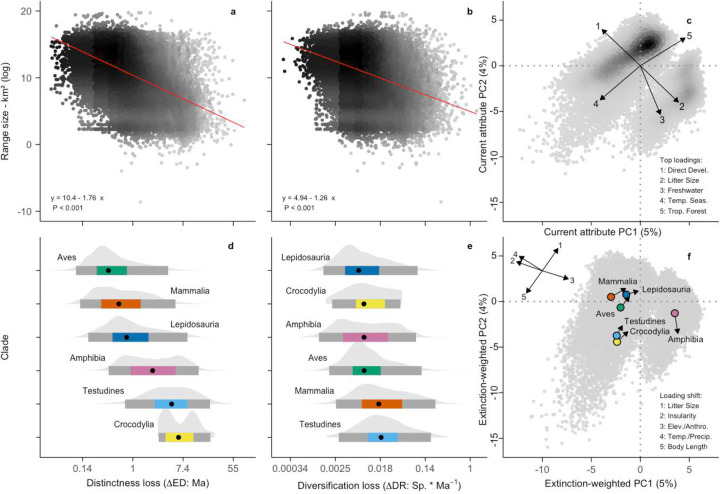
Relationship between trait imperilment and evolutionary outcomes across Tetrapoda. **a, b** Negative relationships between range size (the strongest determinant of pEX) and expected losses of Evolutionary Distinctness (ΔED) and Diversification Rate (ΔDR) at the species level. **c** Principal components of present-day attribute space from 188 variables across Tetrapoda, with major loadings showing the dominant non-phylogenetic attributes structuring global tetrapod diversity. **d, e** Variation in species-level ΔED and ΔDR by clade, showing greatest losses of ancient diversity in turtles and crocodilians and evolutionary potential in mammals and turtles. **f** Extinction-weighted PCA of attribute space showing expected changes due to Anthropocene imperilment, with the factor-loading shifts showing the non-phylogenetic attributes varying within clades that determine the largest differences between imperiled and surviving attribute space and the corresponding clade-level centroid movements along those axes (Extended Data Fig. 9; Supplementary Table 8).

Projected shifts in occupied attribute space weighted by pEX also reveal clade-specific patterns of erosion in trait diversity for attributes that vary within clades (Fig. 3f; Extended Data Fig. 9; Supplementary Table 7, 8). Birds, crocodilians, mammals, and turtles shift away from larger-bodied and less-fecund species. For lepidosaurs, a disproportionate loss of island endemics and lowland tropical taxa and increased representation of species associated with anthropogenic disturbance is the largest change, a pattern that is also partially reflected in mammals. Across amphibians, the relationship of life history and litter size to extinction risk is complex^28,38^, though overall, tropical direct-developing lineages – which are usually smaller – are more imperiled, favoring increased future representation of larger-bodied biphasic species.

Our model highlights branches of the Tetrapod Tree of Life most likely to be pruned, predicting the trajectory of spatial and phylogenetic contractions across assemblages. Under current threats and without intervention, future biotas will likely have reduced representation from numerous major lineages, including large marine, herbivorous, and carnivorous mammals^39^; many larger, phylogenetically distinctive, and island-dwelling birds^33^; most turtles and crocodilians^7^; diurnal lizards^40^; and direct-developing amphibians^38^. Conversely, adaptable generalists with low extinction risk (family median pEX < 0.09, equal to an IUCN Red List status of LC^2^) and higher DR (0.08–0.20 Species * Ma^−1^) will likely dominate recovering biotas, heavily represented from groups like murine rodents, tyrannid birds, colubrid snakes, and hylid frogs. Our analysis does not address the capacity for rapid Anthropocene adaptation or short-term phenotypic plasticity and range shifts to overcome ecological alterations^15^, key areas for future research.

## Shifts in Macroevolutionary Trajectories

The pEX metric reveals how human activity is shifting extinction timelines for extant species, allowing us to assess how Anthropocene pressures are altering these dynamics^1^. We quantify extinction timelines through three complementary approaches, using pEX to calculate species-level estimates of weighted net diversification rate (wDR), tip-specific extinction rates (E/MSY), and aggregated survival probability (1 - pEX). These estimates assume present-day conditions and could shift dramatically in the future as patterns of global change in conservation, land use, climate, and other factors intensify^41–43^. Across our 500-year time span of analysis, expected speciation is negligible as ∑exp(DR_i_ * 0.0005) = 33,284 species^44^; a net gain of three lineages based on macroevolutionary rates.

We first used pEX to adjust DR, estimating the loss of macroevolutionary potential (ΔDR = pEX * DR) and the corresponding extinction-weighted net diversification rate (wDR = DR - ΔDR; Fig. 3 and Extended Data Fig. 9). The median wDR declines by 16% to 0.094 Sp. * Ma^−1^ (0.0017–4.34) from the unadjusted median DR of 0.11 (0.0036–4.61), with a median decrease (ΔDR) of 0.0095 (0.00025–1.33). Estimated ΔDR is highest for turtles, with mammals a close second and exhibiting the greatest interquartile range and extremes (Fig. 3b). In contrast, crocodilians, birds, amphibians, and lepidosaurs show smaller decreases, reflecting their concentrations of species with higher DR and lower pEX. Mammals show disproportionate declines due to high DR and elevated pEX by species, apparently driven by intrinsic traits (e.g., both very large and very small body sizes, each often paired with small geographic ranges) that amplify susceptibility to extrinsic anthropogenic threats like exploitation and habitat change^45^. These factors are less pronounced in other high-DR clades like birds.

Second, treating pEX as the exact probability of extinction after 500 years yields tip-level extinction rates of 43–6,563 E/MSY from μ_i_ = −ln(1 − pEX_i_) / 0.0005^44^, with a median of 126. Third, treating 1 - pEX as the exact probability of survival after 500 years leads to a projected future tetrapod diversity of 28,060–28,252 species from ∑(1 - pEX_i_). This represents a loss of 5,029–5,221 species or ~15% of terrestrial vertebrates (Supplementary Table 7), similar to the 16% projected losses in DR. Expressed in evolutionary terms, this equates to 308 E/MSY. Though we note that pre-historic “background” rates cannot be readily related to one another when time scales differ^1,46^, our estimates substantially exceed commonly-suggested background rates (e.g., 0.1–2 E/MSY; Extended Data Fig. 6a)^46–48^.

Elevated filtering for key traits like body and range size mirrors patterns observed during previous extinction episodes^22,49^, such as the K-Pg event that catalyzed the rise of modern birds and mammals^50,51^. Life-history strategies that are sometimes evolutionary advantageous, such as traits associated with rapid diversification (e.g., direct development in amphibians), ecological stability (e.g., large body size and low fecundity in many birds and mammals), or evolutionary stasis (e.g., the body plans of crocodilians and turtles), often become liabilities during periods of rapid global change^22^. The trait-based filtering arising from anthropogenic global change is reshaping the fitness landscape in potentially predictable ways, possibly driving distinct phases of ecological collapse and subsequent recovery that can be modeled with reference to previous deep-time extinction events^1,22^. A crucial point is that most risk is concentrated in specific lineages across clades, creating initial “pulses” of Anthropocene extinction^16,52^ that should, in theory, level off as resilient lineages begin to adapt over time^1,15,22^.

## Validation and Uncertainty in Extinction Risk

We interpret pEX as the probability of a species reaching ‘EX’ status within 500 years, independently validated using recent empirical assessments of extinction timelines in birds based on changes in IUCN statuses from 1988–2016^31^ in 11,064 species. Our estimates closely match observed species lifetimes, differing by just 2 years for VU birds (1,693 estimated vs. 1,691 observed) and 53 years for all NT tetrapods (2,108 vs. 2,161) when comparing our estimates for all species to the results for birds. The sole discrepancy is that we predict longer persistence for both LC birds (8,953 vs. 3,347 years) and all LC tetrapods (7,457 vs. 3,347), with all other comparisons of waiting times, rates, and pEX for both birds and all tetrapods aligning without significant differences (Extended Data Fig. 4; Supplementary Table 5). In absolute numbers, our estimates of 952–979 bird extinctions over the next ~500 years also fall within the range of previous projected losses ranging from 226–738^52^ to 1,305^16^ species within the next few hundred years. This congruence across different datasets and methodologies supports our approach.

Despite this strong empirical validation, we reiterate that our results are contingent on a variety of factors with both present and future uncertainty^24^ due to data deficiency for many species (Extended Data Fig. 10d) and the unpredictable impact of further global change. Extinction trajectories can shift radically over very short timescales due to unforeseen attribute interactions or increasing anthropogenic impacts, particularly as expected from future climate change and potentially accelerating and shifting our estimates^41,42^. In contrast, intensive interventions can significantly delay, mitigate, or reverse declines for some species^31^. Expert-assessed threat statuses are often based on limited information^52^ and may shift in either direction with new data or assuming wider latitudes for imperilment or recovery. Apparent extinctions that are yet to be formally recognized, rediscovery of species thought to be extinct, discovery of new species, and taxonomic changes affecting known species can substantially impact phylogenetic conservation analyses like ours^53,54^. Finally, future work addressing clade- or region-specific extinction projections based on climate- and land-use change scenarios will provide important complementary insights to our approach based on current threats^40,43^.

## Conclusions

Our findings indicate that Anthropocene extinctions disproportionately impact two evolutionarily successful but ecologically vulnerable modes in tetrapods: rapid bursts of diversification and long-term persistence of distinct lineages, a pattern of potential loss shared with past extinction episodes^22^. High-DR radiations often produce transient lineages that arise and vanish quickly, while high-ED lineages typically exhibit stasis within stable niches. Correspondingly, both orientations are often characterized by specific traits that render them susceptible to global change. These effects are also heterogeneous across the main clades. For example, disproportionate impacts are evident in both recent radiations of terrestrial direct-developing amphibians in narrow tropical niches, but also in ancient groups of turtles, crocodilians, and mammals with large bodies, long lives, and low fecundity. While evolutionary extremes towards rapid diversification or long-term persistence may confer success during periods of macroecological stability, they can also increase susceptibility to rapid fitness declines during short-term ecological disruption. Consequently, the current biodiversity crisis may resemble previous extinction events in producing phylogenetically and spatially homogenized assemblages with reduced attribute diversity^21,38^. These evolutionary vulnerabilities also translate into geographic disparities: tropical islands and mountains face the highest absolute losses, while arid and temperate regions may experience the steepest relative decline due to limited species richness and attribute redundancy^16^. Such patterns arise from the intersection of deep-time evolutionary legacies with rapid and asymmetric ecological disruption. Our framework enables modeling post-extinction recovery^22^ and long-term macroevolutionary shifts^1,15^, while also guiding conservation of ancient diversity and evolutionary potential across the globe.

## Methods

### Phylogenies

**Extended Data Fig. 1 |:**
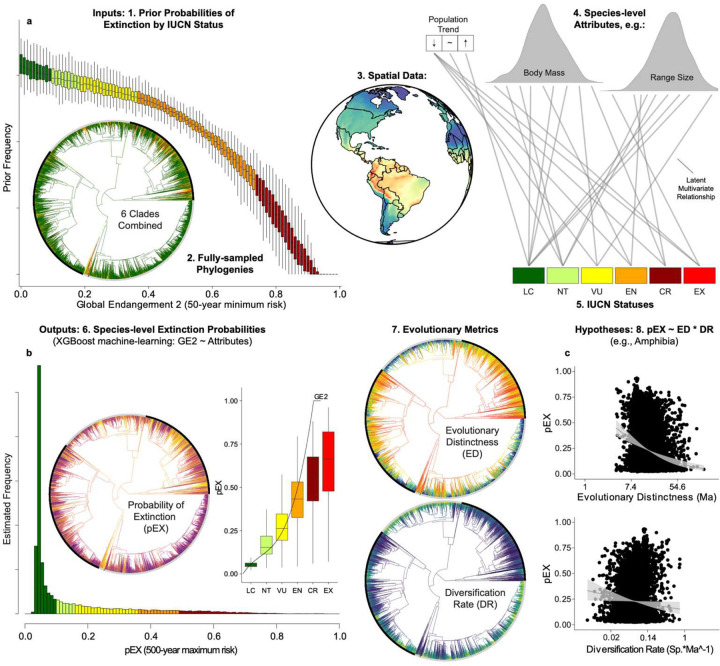
Schematic outline to estimate species-level extinction probabilities (pEX). **a** Inputs combine 6 clade-level phylogenies, 188 species-level attributes (including phylogenetic and spatial filters), 29,827 IUCN statuses, and GE2 50-year minimum extinction risk as prior probabilities for training. **b** XGBoost models trained on the latent multivariate GE2 ~ Attributes relationship and projected onto the original attribute space yield species-level extinction probabilities on a ~50–500-year timescale for all 33,281 tetrapods, along with the macroevolutionary metrics of Evolutionary Distinctness (ED: Ma) and Diversification Rate (DR: Sp. * Ma^−1^). **c** Our primary hypothesis is that pEX disproportionately targets ED and DR across the Tetrapod Tree of Life and the globe, revealing clade- and region-specific patterns.

We combined recent taxonomically complete phylogenetic datasets for amphibians^6^, mammals^8^, squamates^5^, birds^4^, and non-avian archosauromorphs (i.e., crocodilians and turtles)^7^ for a posterior distribution of 10,000 trees for Tetrapoda (Extended Data Fig. 1), consisting of 7,238 amphibians, 5,911 mammals, 9,755 lepidosaurs, 357 turtles, 9,993 birds, and 27 crocodilians. A majority of species (20,612/33,281, 62%) were placed with molecular data – the remainder are imputed from low-level, high-precision taxonomic information^55^.

Previous mammal trees^8^ were estimated using a birth-death branch-length prior, while all other clades used pure-birth (Yule) priors, which could influence analyses relying on unified branch lengths. We re-estimated the mammal phylogeny of 5,911 species as described previously^8^, using the Node-Dated Exponential (NDExp) backbone. We set the relative extinction rate to zero – extinctionpr = fixed(0) – to equal a Yule process. All other parameters were identical. We then grafted all six sets of trees using a unified backbone and timescale.

A single topology *(Amphibia,(Mammalia,(Lepidosauria,(Testudines,(Aves,Crocodylia)))))*; is supported by genomic evidence^56,57^. We used expert consensus estimates from the TimeTree database^58,59^ for uniform priors on node ages. Ages for Sauria, Archosauromorpha, and Archosauria were previously estimated using this strategy^7^. The remaining nodes were Tetrapoda, for which 32 studies yielded a confidence interval of 348–358Ma; and Amniota, for which 30 studies yielded an interval of 294–323Ma^60^. We sampled trees without replacement in order of crocodilians and turtles (pruning the bird)^7^, birds^4^, lepidosaurs^5^, mammals^8^, and amphibians^6^, with the two internal node ages drawn from the given distributions.

We performed PCA on the centered and scaled phylogenetic variance-covariances matrices of 100 random trees using *prcomp_irlba()*in the ‘IRLBA’ package^61,62^. We calculated a maximum root-to-tip node distance of 58, suggesting that a limited number of axes represent most structure (Extended Data Fig. 10a). We used custom R code to calculate the species-level metrics of Evolutionary Distinctness (ED) measuring the independent phylogenetic history in millions of years^34^, and Diversification Rate (DR)^4^ estimating tip-level speciation rate assuming a minimal recent effect of extinction^63^ (Extended Data Fig. 5, 6b). We used the harmonized taxonomies from the previous studies to classify species by family (Extended Data Fig. 9).

### Attributes

We updated the TetrapodTraits database^9^ of 75 fully sampled variables representing 24 attributes for all 33,281 species considered here, including body size, activity time, habitat, biogeography, insularity, ecosystem, environmental preferences, human influence, and threat status. We include 11 new attributes related life history, threat, biome, population trend, and range size^64–82^. We coded three binary reproductive traits for direct development, larval stage, or viviparity and 10 binary variables representing major diet categories plus diet breadth (sum of diet categories), along with two continuous attributes for maximum longevity in years and average brood (or litter or clutch, etc.) size. Another 12 binary variables recorded IUCN threat types plus threat sum, and one ordinal variable tracking recent population trends as declining, stable, or increasing, alongside 14 interval-scaled attributes (BiomeNum_1–14) representing the proportion of each species’ range within WWF biomes (seven forested and seven non-forested). To allow better discrimination of geographic range, we added a measure of range size in km^2^ derived from the original range maps^9^. Further details are given in the Supplementary Methods.

Our threat status data comprise 29,232 species extracted from the IUCN Red List v. 2023–1 (October 5, 2023), with statuses for 605 additional species retrieved from previous IUCN assessments using the same taxonomy^5–8,33,83^ or recent updates^28,84^. These data are incomplete, with missing assessments clustered in mammals and squamates^27,85^, whereas birds have complete statuses^33^, and amphibians were recently updated^28^ to near completeness. Of the 33,281 species in our tree, 29,837 have non-DD assessed statuses. Of the 3,444 without assessments, 2,936 are Data Deficient (‘DD’) and 508 are unassessed (‘UA’). We refer to this as the “Assessed-Only” dataset. We previously imputed statuses – based in part on the phylogenies and traits referenced here – for amphibians^83^ and turtles and crocodilians^7^. Other recent authors imputed squamate statuses^86^ using our trees^5^. We included these in a second set of analyses. Consequently, 2,289 of the 3,444 species with DD/UA statuses have imputed statuses available, leaving 1,093 still DD/UA, mostly in Mammalia. We refer to this as the “Assessed+Imputed” dataset.

The dataset also includes 196 species with assessed IUCN statuses of EX across all clades, ranging from 9 turtles to 113 mammals from the Pleistocene to the 21^st^ century. Inclusion of these taxa should strengthen our model training by giving insight into the attributes most associated with anthropogenic extinction. We generally preserved the strategies described by previous authors for opportunistically including these taxa in the phylogenies^4–8^ and attribute data^9^. Evidence for the former was typically full molecular data when available, limited ancient DNA, or taxonomic constraint. Attribute data similarly ranged from full empirical assessments for recently extinct species, estimates based on fossil or subfossil remains for traits (e.g., body mass), and sparse observations (e.g., subfossil or fossil localities) for geographic distribution.

To account for spatially structured threats, we applied distance-based Moran’s eigenvector maps (dbMEM^87^) using the *vegan* R package^88^. We converted the centroids of each species’ range centroids to an equal-area projection and computed a pairwise Euclidean distance matrix. To focus on ecologically relevant spatial scales, we truncated this matrix at four times the maximum distance from the minimum spanning tree connecting all centroids^87^. Next, we performed a principal coordinates analysis (PCoA) on this truncated matrix to extract Moran’s eigenvectors (spatial filters) and retained only those with positive eigenvalues^89^. From the initial 179 filters (equivalent to >1% of the eigenvalue of the first filter^87^), we selected the first 32 (representing a 5% threshold) for feature selection, while retaining the full set for reference (Extended Data Fig. 10b). This approach captures multiscale spatial structure while minimizing noise and overfitting.

Consequently, our final dataset contained 188 consisting of 32 spatial filters, 58 phylogenetic axes, and 98 species-level variables. These include life-history and other organismal traits, spatial information, and global-change metrics affecting species’ ranges. After feature selection identified the complete dataset as the best predictors, we used these 188 features to estimate the GE2 ~ Attribute relationship to estimate pEX from IUCN status.

### Modeling extinction

**Extended Data Fig. 2 |:**
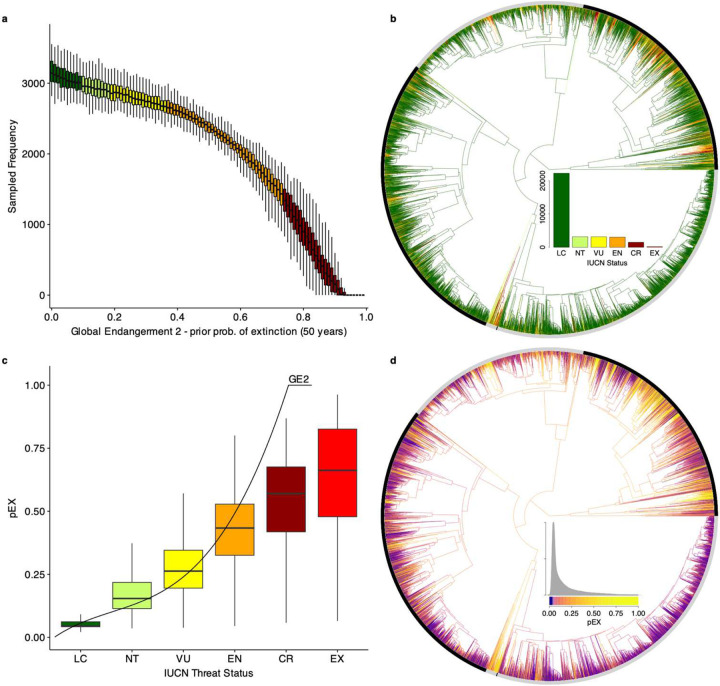
Relationships between GE2 priors on extinction probability, IUCN threat status, and pEX. **a** Sampling distribution of GE2 priors on 50-year extinction probabilities by IUCN threat status, used to generate training data for pEX models as a function of attribute values. **b** Phylogenetic distribution of IUCN threat status across the Tetrapoda Tree of Life, used to sample GE2 extinction priors for each species. **c** Boxplot of pEX by IUCN threat status, overlaid on the GE2 extinction-prior curve. **d** Phylogenetic distribution of species-level extinction probabilities across the Tetrapoda Tree of Life.

We hypothesized that a latent model structure linking species-level attributes to IUCN threat statuses^26,90^ could be used to predict short-term extinction risk – termed “pEX” on the interval [0,1] – from species’ attributes and converted to species-level extinction rates over ecological timescales (Extended Data Fig. 2). We trained machine-learning models^86,91^ to capture the multivariate relationship between attributes as predictors and prior probabilities on extinction risk for IUCN statuses^2^ as responses. Similar approaches have been suggested previously^3,92,93^, but were limited by the lack of species-level datasets and threat assessments^9^.

Recent authors^2^ proposed extinction probabilities for IUCN statuses^34^, which they termed Global Endangerment 2 (GE2). Adopting a 50-year time horizon^3^, they assigned a 97% extinction probability to CR, halving this value for each successive threat level (EN = 48.5%, VU = 24.25%, NT = 12.125%, and LC = 6.0625%). This yielded ranges of LC = 0–9%, NT = 9–18%, VU = 18–36%, EN = 36–72.5%, and CR = 72.5–99.9%. Species assessed as EW and EX were assigned a probability of 100%, with ε (~2.2e-16, machine precision) added or subtracted from 0 or 1 values to avoid absolutes. We follow a similar approach, with some modifications. While GE2 specifies a 50-year interval^2^, the IUCN Red List guidelines specify 100 years, and previous studies have modeled a more conservative 500-year horizon^3,18^. As an improvement over the random-sampling approach in the original EDGE2 study^2^, we treat the GE2 ranges as uniform prior densities on 50-year minimum extinction risk, which we modeled in an attribute -based framework to generate posterior estimates of ~50–500-year maximum risk.

Accordingly, we propose pEX as an estimate of extinction probability across ~50–500 years (Supplementary Table 7), emphasizing the upper bound (i.e., 500 years) which shows strong empirical validation from recent assessments in birds^31^. We estimated the GE2 ~ Attributes relationship using machine learning techniques, implementing extreme gradient boosting for classification/regression trees in the R package ‘xgboost’^25^. We built 100 models with starting values of GE2 drawn from the prior densities given above. We then projected the trained models (GE2 ~ Attributes) back onto the original attributes to generate optimized pEX values. For Data Deficient (DD) and unassessed (UA) species, we predicted pEX based on their attributes and imputed their threat status using the GE2 thresholds^2^ (Extended Data Fig. 10d).

We thereby produced a harmonized set of statuses and pEX across all 33,281 species in the phylogeny. We first used only the 29,837 species with assessed IUCN statuses, the “Assessed-Only” model yielding “pEX1,” with pEX and status for the 3,444 DD/UA species being predicted afterwards. We then repeated the analysis using the 29,837 assessed statuses and the 2,289 previously imputed statuses, predicting pEX and status for the 1,093 DD/UA species in the prediction set. This is the “Assessed + Imputed” model, yielding “pEX2.” Both pEX and variable importance show strong overall concordance (i.e., *r* > 0.99). Small positive residuals for some lower-pEX species in Dataset 1 show higher predicted risk in Dataset 2 (Extended Data Fig. 10e, f). We hypothesize that previous imputations were skewed towards higher status levels by less-flexible modeling, smaller datasets, and fewer predictors. Beyond this, we do not analyze pEX2 or Dataset 2 further but provide them for comparison if desired.

### Model fitting

We first optimized hyperparameters for the training dataset including all features using the R package ‘ParBayesianOptimization’^94^, with 100 initial samples and 10 iterations, after which the ‘utility’ metric – used to guide exploration of hyperparameter space – declined to ~0, indicating convergence. We only used assessed statuses for hyperparameter training, subsampled to 20% for computational feasibility. For the ‘score’ metric, we maximized negative *rmse*, the standard metric for the *reg:logistic* classifier, with expected improvement (‘ei’) as the acquisition function. We evaluated 11 hyperparameters related to learning rate (*eta*), tree topology (*max_depth*, *max_delta_step*, *min_child_weight)*, subsampling (*subsample, colsample_by_tree, colsample_by_level, colsample_by_node*), and regularization (*gamma, lambda* and *alpha*). Finally, we set *base_score* to the proportion of species classified as extinct (EX).

We performed 25 replicates, selecting the optimal hyperparameter set based on the lowest median *rmse*, and averaging the resulting estimates for the pEX models. We assessed predictive testing accuracy on a hold-out dataset and used this as our final metric to choose among feature sets. We tested feature selection using five distinct strategies. We re-tuned hyperparameters individually for each set. The five sets were (i) all features, (ii) an all-relevant set via random forests using the ‘Boruta’ package^95^, (iii) Boruta using xgboost yielding a minimal-optimal set, (iv) the minimal-optimal set of (iii) and all pairwise products thereof, and (v) the minimal-optimal set of (iii) and all arithmetic combinations thereof.

The dataset containing all predictors performed the best, returning the lowest *rmse* for the hold-out testing data (Extended Data Fig. 10c). Consequently, we used the full set of features to optimize our final sets of 100 pEX models for the initial GE2 ~ Attribute model training. We then drew a random value of GE2^2^ from the range implied by the status available for each species. That status will have been assessed or imputed, based on which species and dataset (“Assessed-Only” for pEX1 or “Assessed + Imputed” for pEX2) was under consideration. The randomly drawn GE2 values formed the response variable for the logistic regression models optimized in xgboost, using the full attribute dataset as the predictors.

**Extended Data Fig. 3 |:**
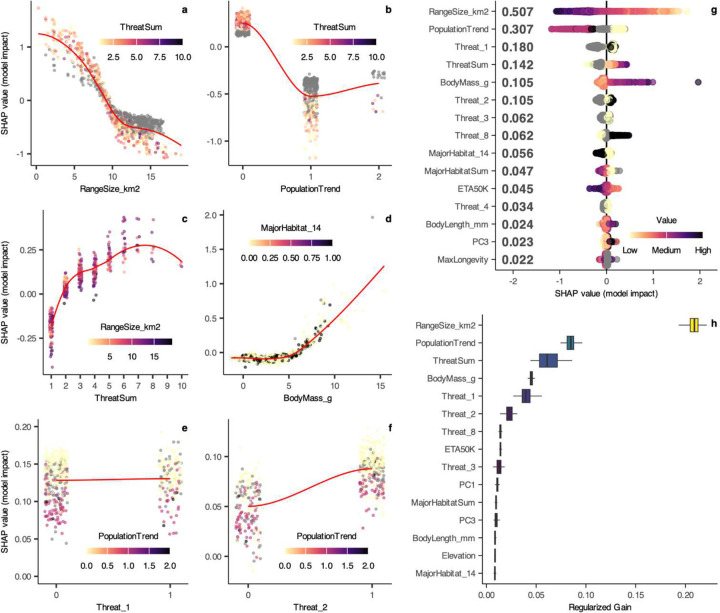
Variable importance for attributes contributing to pEX. SHAP plots showing model impact of **a** range size, **b** population trend, **c** sum of threats, **d** body mass (g), **e** residential and commercial development, and **f** agriculture & aquaculture, with the primary interactions for each variable indicating secondary impacts of sum of threats, population trends, the first phylogenetic axis separating amphibians from other tetrapods (Extended Data Fig. 10a), and range size. **g, h** SHAP summary plots and relative gain estimates showing the model impact of the 15 most important variables contributing to the XGBoost models.

For model training, we implemented ‘hist’ trees, an 80/20 train/test split with 5-fold cross-validation, early stopping after 50 rounds of non-improvement up to 5,000 rounds, and estimated variable importance using several metrics (Extended Data Fig. 3). For each of the 5-fold ensemble models, we recorded variable importance as the regularized ‘Gain’ metric, which quantifies each feature’s contribution to prediction accuracy. To understand interactions^96,97^, we calculated importance using the ‘SHAPforxgboost’ package^98,99^, where SHAP plots quantify feature-response curves highlighting interactions with other features. Given their complexity, we limited the SHAP analysis to a single model for visualization purposes (Extended Data Fig. 3).

### Empirical validation

Recent authors^31^ projected empirical extinction timelines for 11,064 bird species based on observed movement between IUCN statuses from 1988–2016, which they expressed as mean species lifetimes (*T*) in years with and without conservation, the latter excluding decreases in status resulting from active intervention. From their estimates of *T*, we calculated μ = 1 / *T* and pEX = 1 - exp(-μ * 0.005)^44^, and converted our pEX estimates into μ and *T* for comparison. Their dataset treated EW as a separate status for which they projected lifetimes, and used 0/Inf/1 for *T*, μ, and pEX across all EX species, while our models lump EW/EX taxa and estimate those parameters. We compare our EW/EX group to their EW group, all labeled “EX” for reference.

**Extended Data Fig. 4 |:**
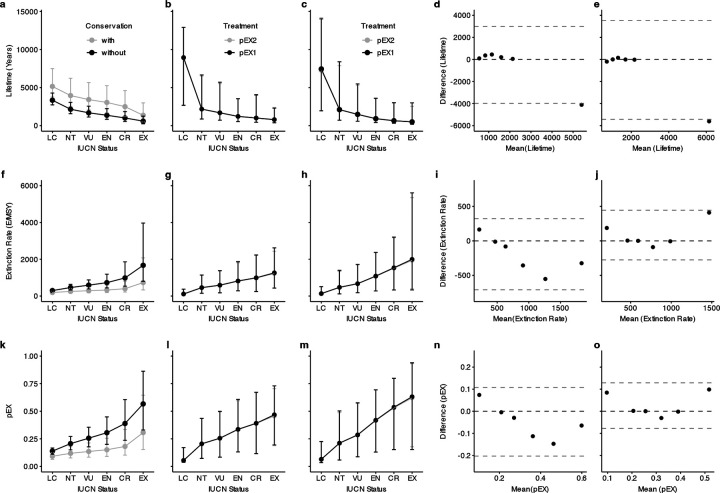
Empirical validation of pEX in comparison to IUCN status changes in 11,064 species of birds from 1988–2016 yielding species’ lifetimes, extinction rates (E/MSY), and 500-year extinction probabilities (pEX). Species-level lifetimes (years), extinction rates (E/MSY), and pEX across IUCN statuses estimated **a, f, k** empirically for 11,064 birds with and without conservation intervention between 1988–2016, **b, g, l** for the 9,993 bird species in our phylogeny, and **c, h, m** for all 33,281 tetrapod species in our phylogeny. Comparing the empirical and estimated datasets using Bland-Altman plots, where dashed lines indicate thresholds of *P*<0.05 for mean differences, reveals significance only for the longer estimated lifetimes of LC species in our dataset for **d** birds and **e** all tetrapods relative to the empirical data. Species’ lifetimes for all other threat statuses and for **i, j** extinction rates and **n, o** pEX across birds and tetrapods are congruent at *P*>0.05 for both pEX1 and pEX2.

We compared the without-conservation species lifetimes^31^ to our pEX1 estimates for both birds and all tetrapods using a Bland-Altman plot^100^ of the mean versus the difference of species lifetime, extinction rate, and pEX (Extended Data Fig. 4; Supplementary Table 5). The only significant difference is a longer mean lifetime (~4,100–5,600 years) estimated for LC species in both groups using our pEX1 dataset. For the other statuses, non-significant differences in estimated versus observed lifetime range from 2–194 years in birds and 53–450 for all tetrapods. The estimated extinction rates (E/MSY) and pEX for all statuses are not significantly different for birds or tetrapods. Congruence of these estimates across different datasets and methodologies strongly support empirical applicability of our models to contemporary extinction timelines.

### Spatial Analysis

We mapped species-level attributes to examine global macroevolutionary and macroecological patterns of diversification and extinction^101^. For assemblage-level estimates, we used spatial intersections of species range maps with an equal area grid cell of 110×110 km to derive species lists for each grid cell for each clade^9^. We then computed the mean log_10_(ED) and log_10_(DR), geometric mean of pEX, and log_10_(range rarity, defined as the sum of the inverse range sizes of all species present in a grid cell^102^) across species within each assemblage (Extended Data Fig. 8).

To account for potential richness dependence of assemblage-level metrics^103–108^, we generated null expectations for ED, DR, pEX, and range rarity for each assemblage and calculated standardized effect sizes to quantify deviations from these expectations. We computed the null distribution of assemblage-based metrics by randomizing species names in the attribute table for regional species pools while holding the internal structure of the species-by-grid-cell matrices (e.g., species-occurrence frequency, species richness, and co-occurrence patterns^109^) constant.

We defined species pools using biogeographical realm and biome classifications^78^. Each 110×110 km grid cell was assigned to its dominant realm-biome combination (bioregion^105^), and spatial intersections between grid cells and species ranges^9^ were used to calculate the proportion of each species range within each bioregion. We included species occupying ≥10% of the bioregion or if the bioregion covered ≥10% of the range, allowing associations with multiple bioregions. We ran 1,000 null models for each bioregion, using the respective grid cells and species pool, and then calculated the standardized effect size (SES = observed - mean[null] / SD[null]) for each assemblage-level metric. SES values below −1.96 or above +1.96 indicate metrics significantly lower or higher than expected, respectively, given species richness.

**Fig. 4 |:**
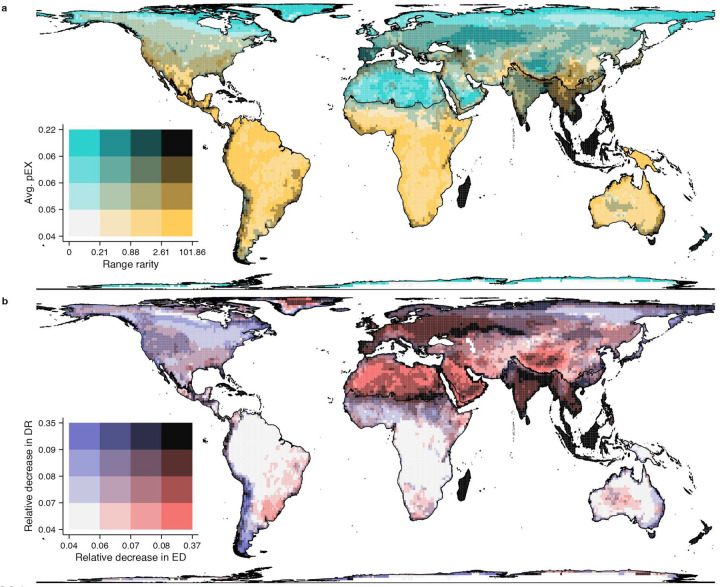
Distribution of extinction risk, endemism, and macroevolutionary metrics under current global-change regimes. **a** Geometric mean of extinction risk (pEX) and total range rarity (∑ 1 / range size [km^2^] of all species occurring in a cell) across global assemblages of 110×110km grid cells, showing coincidence of extinction risk and local endemism. **b** Expected change in Evolutionary Distinctness (ED) and Diversification Rate (DR) across assemblages, showing the relative decrease of original assemblage values after accounting for pEX (Extended Data Fig. 8). Color scales in the bivariate choropleth were based on quartile intervals of the respective variable.

Finally, we intersected the species range maps and our 110×110 km grid-cell scheme to identify changes in assemblage-level metrics. Specifically, we computed the relative change in ED and DR due to projected species loss, with relative decrease in ED calculated as the sum(ED * pEX) / sum(ED) per assemblage, and the relative decrease in DR obtained as mean(DR * pEX) / mean(DR). We divided assemblage-level values for each metric into quartiles, yielding four intervals per variable. We constructed a bivariate color scheme by combining these intervals, assigning distinct colors to each of the 16 (4 × 4) possible combinations. We mapped patterns of relative decrease in ED-DR and overlaps in log_10_(range rarity) and geometric mean pEX (Fig. 4).

### Macroevolutionary trends

**Extended Data Fig. 5 |:**
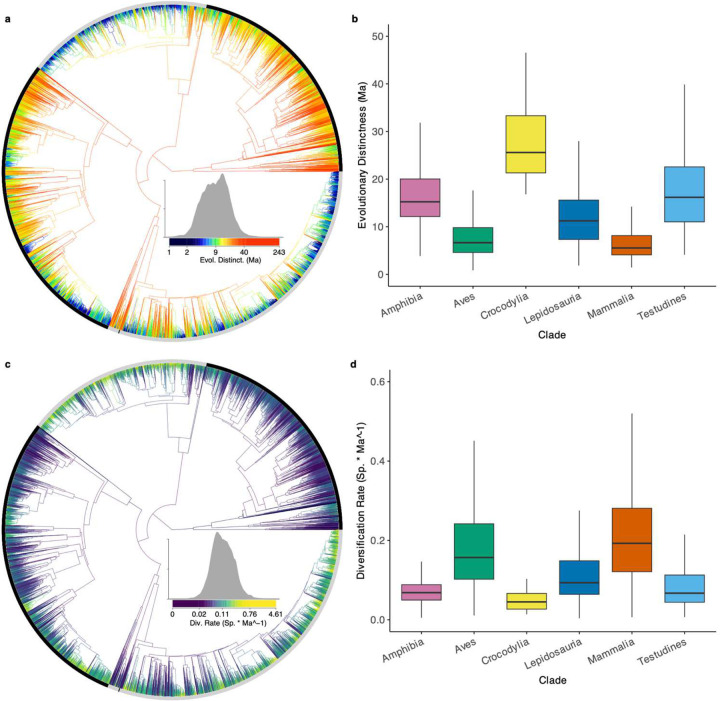
Distribution of ancient diversity and evolutionary novelty across the Tetrapod Tree of Life. **a, c** Phylogenetic distribution of Evolutionary Distinctness (ED: Ma) and Diversification Rate (DR: Sp. * Ma^−1^) across the 33,281 species in our phylogeny. **b, d** Boxplots of ED and DR across the six major tetrapod clades.

**Extended Data Fig. 6 |:**
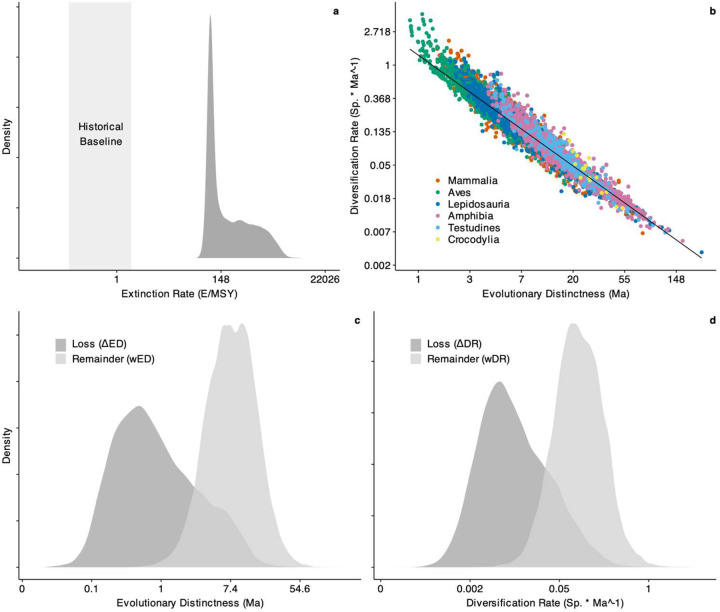
Impact of Anthropocene extinction on ancient diversity and evolutionary novelty in Tetrapoda. **a** Distribution of estimated species-level extinction rates (E/MSY) based on pEX compared to commonly reported historical baselines of 0.1–2 E/MSY. **b** Relationship between Evolutionary Distinctness (ED) and Diversification Rate (DR) as tip-level metrics, showing strong correlation but substantial residual variation among species. **c, d** Estimated species-level impacts of pEX on ED and DR, showing expected loss (ΔED/DR) and extinction-weighted remainder (wED/DR) after 500 years.

**Extended Data Fig. 7 |:**
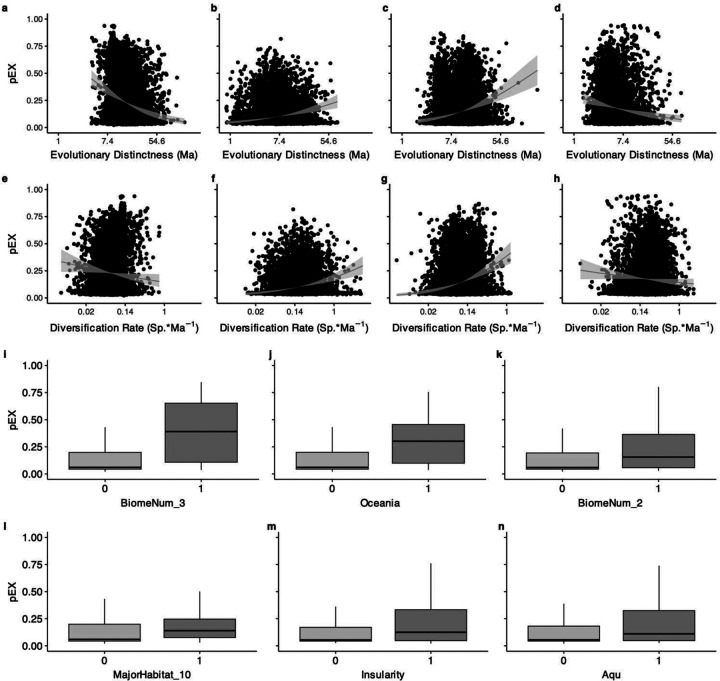
Variation in pEX related to ED, DR, and major attributes. Significant clade-level relationships (Supplementary Table 3) for μ and φ of pEX ~ ED * DR in **a, e** amphibians, **b, f** birds, **c, g** lepidosaurs, and **d, h** mammals. Major binary attributes showing >0.05 median difference in pEX between states with >200 species in each state are **i** tropical & subtropical coniferous forests, **j** occurrence in Oceania, **k** tropical & subtropical dry broadleaf forests, **l** marine environments, **m** island endemics, and **n** aquatic environments.

An overarching question is whether present-day extinction risk disproportionately affects high- or low-ED/DR lineages^10,34^ (Extended Data Fig. 5–7), which was previously limited by the lack of species-level extinction rates. We implemented beta regression models assessing pEX as a function of ED, DR, and Clade (including all main effects and interactions) using the R package ‘betareg’^110^. We tested all possible models and assessed fit using AIC. Each model had up to 6 main effects (i.e., μ and φ ~ ED, DR, and Clade) and two operators (+ or *), yielding 14 sets of predictor combinations each for μ and φ and 196 possible models with up to 48 parameters including the null models of μ and φ ~ 1. The full 48-parameter model (model_196) received overwhelming support (ΔAIC = 102; Supplementary Table 2).

We then performed clade-specific Bayesian modeling using ‘brms’ to estimate parameter uncertainty through credible intervals (Supplementary Table 3). We fitted each model using 4 chains with 2,000 iterations and 1,000 warmup samples, yielding bulk and tail ESS > 1,000 for all parameters. We placed priors of a normal (0,1) distribution for slopes (replacing default uniform priors) and the default t (3, 0, 2.5) distribution for intercepts. We compared models using the WAIC() function, selecting the model that minimized the expected log pointwise predictive density (*elpd*), favoring the simpler model when values were comparable. Finally, we re-estimated the full model_196 in ‘brms’ to obtain Bayesian credible intervals for all 48 parameters. We did not implement phylogenetic versions of our ‘brms’ models despite the availability of methods to incorporate phylogenetic covariance as a random effect^111,112^.

First, the well-known phylogenetic clustering of extinction risk in tetrapods^5,83,113^ suggests that shared evolutionary history often drives the imperilment of entire clades, a conclusion that would be obscured by ‘phylogenetic correction.’ Second, since ED and DR, are derived from the same phylogenetic data and the phylogeny is also used as a predictor for pEX, additional correction would introduce undesirable circularity. Third, pEX represents an instantaneous estimate of extinction probability that can change dramatically over short timescales, making it poorly suited for models assuming heritability – even if the traits influencing pEX themselves are heritable. Fitting pEX ~ ED * DR using independent contrasts shows directionally congruent main effects and interactions but extremely small effect sizes (*r*^*2*^ ~ 0), confirming our assessment that such models are likely redundant and uninformative (Extended Data Fig. 10g–j; Supplementary Table 6). Finally, we estimated 7 binary attributes for which median pEX differed by >5% between species with and without the condition (Supplementary Table 4), and factor-loading shifts between extinction- and survival-weighted attribute spaces (Supplementary Table 8).

## Extended Data

**Extended Data Fig. 8 |:**
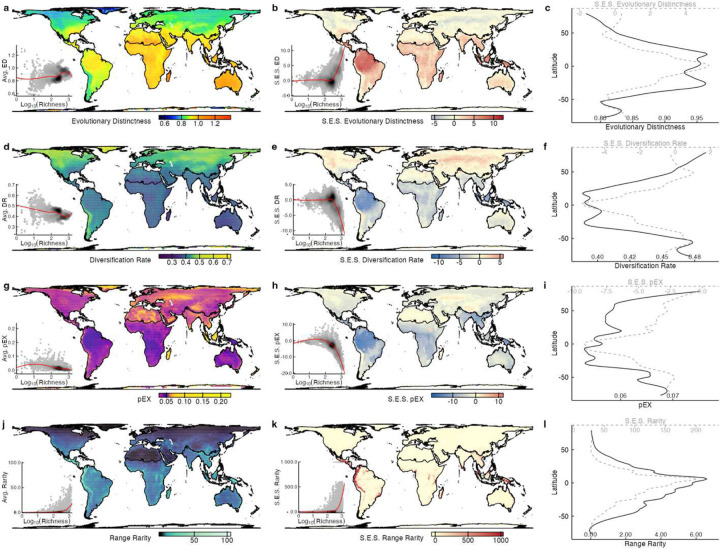
Global distribution of species-level ancient diversity, evolutionary novelty, extinction risk, and range rarity. **a, d, g, j** Assemblage-level values of mean Evolutionary Distinctness (ED), mean Diversification Rate (DR), geometric mean pEX, and log_10_(range rarity) calculated over 110×110km grid cells. **b, e, h, k** Richness-independent values for the same metrics (ED, DR, pED, and range rarity) derived from a null model of randomly shuffled taxon names within the same biogeographic realm-biome species pool. **c, f, i, l** Raw and richness-independent values of each metric (ED, DR, pED, and range rarity) by latitude.

**Extended Data Fig. 9 |:**
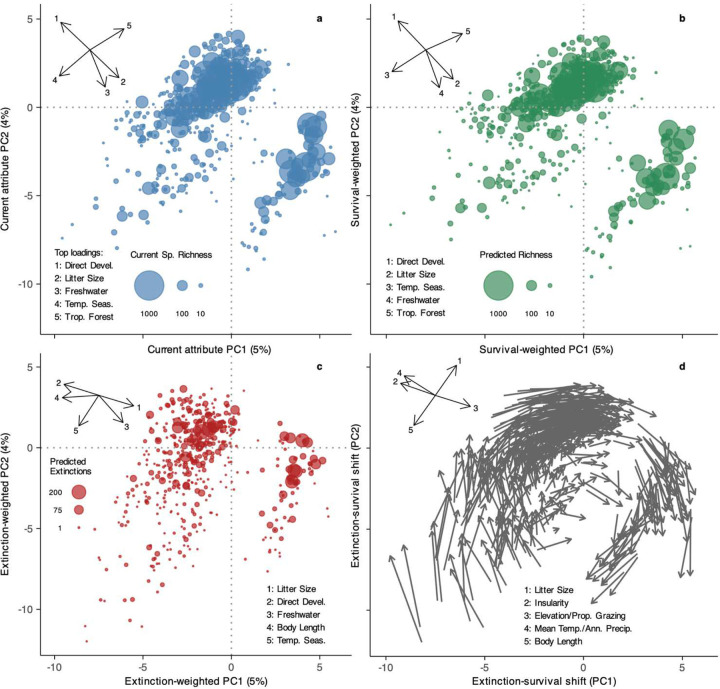
Attribute space of Tetrapoda summarized at the family level (*n* = 509) based on PCA of 188 attributes, showing projected impacts of extinction. **a** Attribute space summarized by family and scaled by species richness for the present day. **b** Future projections weighted by species-level survival probability (1 - pEX) after 500 years. **c** Extinction-weighted projections after 500 years. **d** Individual shifts of the 509 families from the present to the future after weighting by survival versus extinction. The inset rays show the loadings of **a–c** the major non-phylogenetic factors structuring attribute diversity across clades and **d** the loading shifts in major factors related to extinction risk within clades.

**Extended Data Fig. 10 |:**
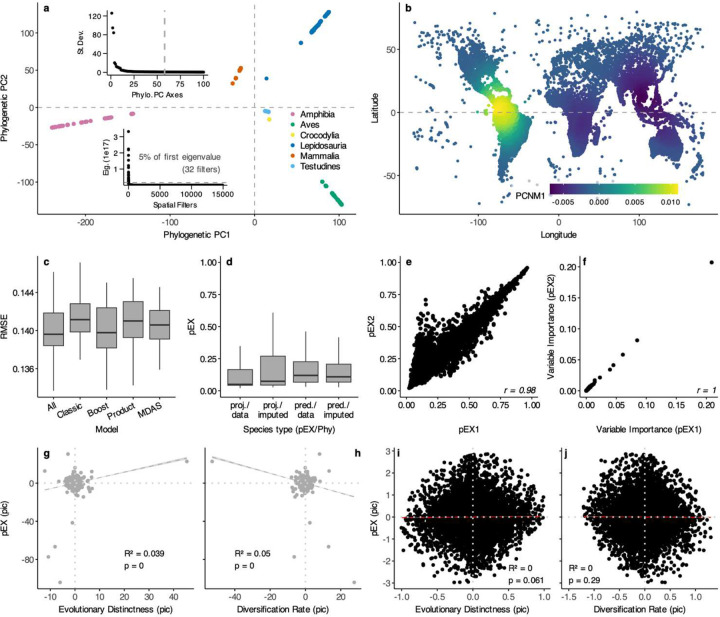
Model parameters, feature selection, and phylogenetic comparison of pEX estimated using XGBoost. **a** Phylogenetic PC axes from our 33,281-species phylogeny showing separation of the six major clades, with inset scree plots showing selection of 58 PC axes and 32 spatial filters. **b** Spatial filter showing autocorrelation of species centered in northern South America versus Indomalaysia – successive axes show differentiation of other regions. **c** Hold-out testing accuracy for 5 sets of raw and engineered features, showing best fit for the 188 attributes. **d** Projected and predicted pEX values for species classified by presence of IUCN statuses and molecular data (proj./data), statuses without molecular data (proj./imputed), no status with molecular data (pred./data), and no status or molecular data (pred./imputed). **e, f** Relationship between pEX1 (assessed-only IUCN statuses for GE2 training data) and pEX2 (assessed + imputed statuses) and variable importances (regularized gain) for the 188 attributes. **g-j** Phylogenetic independent contrasts of Evolutionary Distinctness, Diversification Rate, and pEX with and without outliers, showing minimal effect sizes across most species.

## Supplementary Files

This is a list of supplementary files associated with this preprint. Click to download.

- tetrapod1.0SupplementaryInformation3September2025.pdf

**Supplementary Information** The online version contains supplementary materials available at $XXX.

## Data Availability

All data generated and analysed in the current study are available via http://www.vertlife.org/ and Zenodo repositories 10.5281/zenodo.10530617 for the TetrapodTraits attribute data and 10.5281/zenodo.16415110 for the new pure-birth mammal phylogenies, full TetrapodTrees phylogenetic dataset, and code for the tree assembly and species-level metrics.

## Code Availability

All R code and scripts used for the pEX analyses are available at Zenodo repository 10.5281/zenodo.16787074.
